# The TRACK-MS Test Battery: A Very Brief Tool to Track Multiple Sclerosis-Related Cognitive Impairment

**DOI:** 10.3390/biomedicines10112975

**Published:** 2022-11-18

**Authors:** Daniela Taranu, Hayrettin Tumani, Jill Holbrook, Visal Tumani, Ingo Uttner, Patrick Fissler

**Affiliations:** 1 Department of Neurology, Faculty of Medicine, Ulm University, D-89071 Ulm, Germany; 2 Department of Psychiatry, Faculty of Medicine, Ulm University, D-89071 Ulm, Germany; 3 Psychiatric Services Thurgau, CH-8596 Münsterlingen, Switzerland; 4 University Hospital for Psychiatry and Psychotherapy, Paracelsus Medical University, A-5020 Salzburg, Austria

**Keywords:** cognition, multiple sclerosis, cognitive assessment, disease progression, tracking, test quality criteria, BICAMS, TRACK-MS

## Abstract

Tracking cognition in patients with multiple sclerosis (MS) is important for detection of disease progression but it is often not performed in routine settings due to time constraints. This exploratory cohort study aims to develop a very brief repeatable tracking tool with comparable test quality criteria to the current gold standard, the Brief International Cognitive Assessment for MS (BICAMS). The study included 88 participants (22 healthy controls, 66 MS patients) who were examined at baseline and at one-year follow-up. As a validity criterion for the six administered cognitive tests, we assessed the difference between MS patients and HC, and the correlation with MS-related disability. Combining the two tests with the highest validity—the Controlled Oral Word Association Test and Symbol Digit Modalities Test—yielded an administration time of 5 min. Comparing this new TRACK-MS test battery with the 15 min BICAMS indicated that TRACK-MS showed larger differences between MS patients and healthy controls, a higher correlation with MS-related disability, smaller practice effects, and a good test–retest reliability. We provide evidence that TRACK-MS, although faster to administer, showed at least comparable quality criteria as the BICAMS. As the study was exploratory, replication of these results is necessary.

## 1. Introduction

Cognitive impairment is a frequent symptom in multiple sclerosis (MS), found in 40–70% of patients [1,2,3]. Organized in multiple cerebral networks and pathways, cognition can be grouped into various domains such as executive functions, learning and memory, attention, visuospatial, perceptual and social functions [4,5]. As recent studies demonstrate, cognitive functions are interrelated with other behavior-relevant factors such as emotion and affect [6,7]. Complex metabolic and neurohormonal pathways mediate these functions and play a role in the development of various psychiatric and neurological diseases [8,9].

Jean-Martin Charcot was the first to describe cognitive problems in MS, but it was not until 1991 that S. Rao brought renewed attention to the topic, aiming to reveal the precise mechanisms of MS-related cognitive impairment in the hope of developing effective treatments [10]. Although all cognitive domains can be altered in MS [10,11,12,13,14,15,16,17], the extent and severity of cognitive deficits are quite variable, and depend on many factors, including the type of MS [18]. In general, secondary-progressive MS (SPMS) patients have more pronounced cognitive impairment [19], at least in part because the severity of symptoms is directly associated with the longer duration of disease in these patients. This contrasts with patients who have a relapsing MS course, or patients with primary progressive MS, which is often characterized by an onset late in life [20,21,22]. Cognitive function may be affected even before the first local symptom of MS occurs [23], and reliable assessment of cognition as well as regular surveillance in the course of the disease is necessary. Even subtle cognitive impairment can cause deterioration in patients’ quality of life and negatively affect their participation in work and social activities [24,25,26,27,28,29]. Moreover, monitoring changes in cognition in patients with a clinically isolated syndrome or radiologically isolated syndrome may contribute to identifying those at risk of MS [30,31,32]. Tracking cognitive change helps to predict the potential clinical course and to assess the effect of medical treatment and rehabilitation [33,34,35]. Additionally, in some patients cognitive impairment is the only symptom of MS, so assessing this impairment regularly is the only way to track disease activity [35,36,37]. However, cognitive assessment in routine clinical practice is still limited because of time constraints [38] and often also because of an unfamiliarity with and insufficient training of the testing procedures. Attempts to assess the cognitive status of MS patients with simple screening tests such as the Mini-Mental Status Examination [39] or the Montreal Cognitive Assessment [40] proved to be unsuitable, since these tools are unable to pick up subtle deficits, and the results are dependent on age and educational level [31,41,42]. A very brief cognitive tracking tool is needed that is valid, reliable, time efficient, easy to administer, and has no practice effects [43].

An international panel of experts recommended the use of the Brief International Cognitive Assessment for Multiple Sclerosis (BICAMS) as a neuropsychological screening tool in order to improve the clinical care of patients with MS [44,45,46]. The test is made up of three subtests: the California Verbal Learning Test II (CVLT-II); the Symbol Digit Modalities Test (SDMT); and the Brief Visual Memory Test-Revised (BVMT-R) [46,47]. Assessment using this battery takes about 15 to 20 min, and it can also be performed in smaller facilities and practices without special neuropsychological expertise [46]. In the German language version of the BICAMS, the Verbal Learning and Memory Test (VLMT) is included instead of the CVLT II, and this version is designated as the BICAMS-M [45]. The BICAMS successfully identified impaired cognitive function in adults with MS compared with healthy controls [44]. Deficits in MS patients were observed in all three cognitive domains assessed by the BICAMS: information processing speed, immediate verbal recall memory, and immediate visual recall memory [44].

Using two of the three BICAMS tests—the SDMT and BVMT-R—provided very similar test–retest reliability and validity in comparison with the complete BICAMS in terms of identifying cognitive impairment in MS patients. This abbreviated test procedure is known as the BICAMS-short [48]. If the BICAMS-short is considered too time-intensive, yearly administration of the SDMT is recommended instead [49]. The SDMT is considered to be the most valid single test, with a specificity of 0.60 and a sensitivity of 0.91 [49]. In addition, decline in the SDMT is associated with decline in clinical status [50]. The SDMT was also shown to be the best predictor of employability in studies including multiple cognitive or motor performance tests [50,51]. However, the SDMT is not sufficient to determine impairment in other cognitive domains [52].

Overall, despite the demonstrated clinical utility of the BICAMS and its subtests, research on cognitive testing for MS patients remains sparse [53]. Data are lacking as to whether, in comparison with the BICAMS, other cognitive tests may be more valid, reliable, and time-efficient, and have smaller practice effects [54]. Combining other cognitive tests may yield a shorter battery with similar or better test quality criteria. In the current exploratory cohort study, six cognitive tests were administered (three included in the BICAMS) with the aim of identifying a shorter test battery than the BICAMS that has better or similar test quality criteria, including validity, test–retest reliability, and practice effects.

## 2. Materials and Methods

### 2.1. Study Population

Participants were recruited from the MS outpatient clinic in Ulm, Germany for the study and largely came from Ulm and the surrounding area. In total, 66 MS patients (primary progressive MS: *n* = 22; secondary progressive MS: *n* = 22; relapsing-remitting MS: *n* = 22) and 22 healthy controls (HC) were examined at baseline and after 12 months of follow-up. We used the STROBE cohort reporting guidelines [55]. Inclusion criteria were as follows: Age between 18 and 85, clinically proven MS (primary progressive, secondary progressive, relapsing-remitting types), good communication abilities to conduct the assessment, to understand the purpose of the examination and to provide their informed consent. Exclusion criteria were as follows: Another illness, if it was expected to impair the clinical assessment of MS symptoms, or a history of severe depression or another severe psychiatric disorder.

### 2.2. Procedures

Participants signed a written informed consent form, and the study was approved by the Ethics Committee of Ulm University in 13 September 2016 (File number 157/16). The patients did not receive any economic compensation for their participation. All assessments were performed by a single clinical neurologist (DT).

At baseline and at 12 months, all participants underwent a comprehensive neurological status examination as well as a detailed neuropsychological assessment, and were administered the Expanded Disability Status Scale (EDSS) [56]. For the neuropsychological assessments, we included tests that assessed cognitive domains typically affected in MS: the Controlled Oral Word Association Test (COWAT) for verbal fluency [57], Verbal Learning and Memory Test (VLMT) for verbal episodic memory [45], BVMT-R for visual episodic memory [45], Symbol Digit Modalities Test (SDMT) for processing speed [45], Paced Auditory Serial Addition Test (PASAT) for working memory [58], and the Block Design Test (BDT) for visuospatial reasoning [59] (for a detailed description, please refer to the Supplementary Materials section). Although not frequently used in MS patients, the COWAT was included, because it is short and comprehensively validated and therefore qualified as a potential tracking tool. The sequence of the individual assessments was performed in the same order for all test participants and the tests for individual patients were administered at the same time of day at baseline and follow-up.

### 2.3. Statistical Analyses

Statistical analyses were performed using SPSS version 26 (IBM Corp., Armonk, NY, USA). Statistical significance was set at alpha level < 0.05. Group differences were examined with an ANCOVA at baseline. Covariates included age and education. Additionally, effect sizes (ES) for group comparisons (MS vs. HC) were defined as group differences at baseline divided by baseline standard deviation (z-standardization). Multiple regression analyses were performed to assess the relationship of MS-related disability with all outcome variables. Standardized beta (β) was reported as an effect size measure. Three composite scores were constructed by averaging z-standardized scores: (1) TRACK-MS test, composed of the COWAT and SMDT scores; (2) the BICAMS-short, composed of the SDMT and BVMT-R scores; and (3) the BICAMS, composed of the VLMT, BVMT-R, and SDMT scores. 

The practice effect of each test was examined using two-tail paired *t*-test. Test–retest reliability over the period of 1 year was assessed using correlation analysis between pre- and posttest. The size of the practice effect was calculated and determined by Cohen’s criteria (Cohen, 1988): 0.21–0.49 indicating small; 0.50–0.79, medium; and over 0.80, large effect size.

The number of participants was calculated with a power analysis based on the primary aim of the study (assessing differences between different MS types and healthy controls in baseline and 1-year change of cerebral symptoms including cognitive impairment, fatigue and psychopathology, for details see [60]). The exploratory analysis of the study reported in this manuscript, has a power of 80% to detect (i) small to medium true effect sizes (*r* = 0.29) for associations (e.g., link between cognitive performance and MS-related disability), and (ii) medium to large effect size (Cohen’s *d* = 0.70) for differences between patients with MS and healthy controls, assuming an α-error of 0.05.

## 3. Results

In total, 88 people were included in this study (22 HC and 66 patients with MS; see Table 1 for demographics and clinical characteristics). The raw scores of the cognitive test results for baseline and for the follow-up after one year are presented in Table 2.

**Figure 1:**
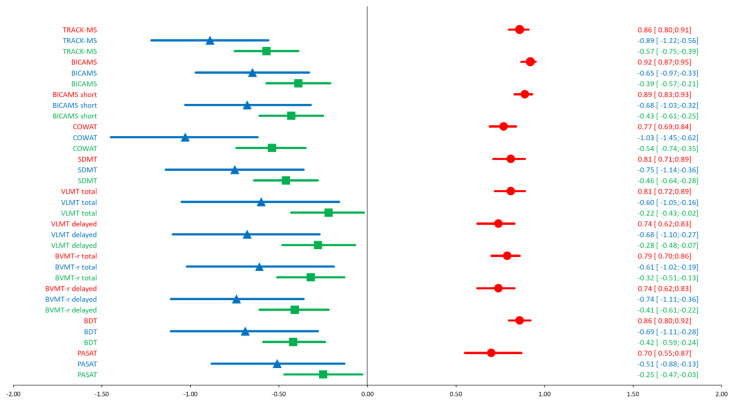
Reliability and validity: Association of baseline cognitive test scores to its re-test scores (test-retest reliability) and to MS-related disability (EDSS, validity). Group difference in baseline cognitive test scores between MS patients and healthy controls (validity). ■ Comparison MS patients vs. healthy controls ▲ Correlation with EDSS ● Test–retest reliability. Abbreviations: BICAMS: Brief International Cognitive Assessment in Multiple Sclerosis; COWAT: Controlled Oral Word Association Test; SDMT: Symbol Digit Modalities Test; VLMT: Verbal Learning Memory Test; BVMT-r: Brief Visual Memory Test-revised; BDT: Block Design Test; PASAT: Paced Auditory Serial Addition Test.

HC had more years of education (*p* = 0.03). In all individual cognitive tests, MS patients had lower scores than HC (*p* < 0.04), but only the COWAT showed a large effect size (ES = −1.03 *SD*, CI: −1.45 to −0.62, *p* < 0.001), followed by the SDMT (ES = −0.75 *SD*, CI: −1.14 to −0.36, *p* < 0.001) (see Figure 1). In addition, all scores correlated with MS-related disability (*p* < 0.03), but only the COWAT was associated with a large effect size (*r* = −0.54, CI: −0.74 to −0.35, *p* < 0.001), followed by the SDMT (*r* = −0.46, CI: −0.64 to −0.28, *p* < 0.001) (see Figure 1). The SDMT, BDT, and VLMT tests had a good 1-year retest reliability (r ≥ 0.80), while the BVMT, COWAT and PASAT tests showed an adequate reliability (r ≥ 0.70) (see Figure 1).

Comparing the so-called TRACK-MS that combined the two most valid tests (COWAT and SDMT) with the current gold standard of the BICAMS, TRACK-MS showed a larger difference between patients with MS and HC with a large effect size (ES_TRACK-MS_ = −0.89, CI −1.22 to −0.56, *F*(1, 84) = 21.6, *p* < 0.001, ƞ2 = 0.21). The BICAMS demonstrated medium effect sizes (ES_BICAMS_ = −0.65, CI: −0.97 to −0.33, *F*(1, 84) = 13.7, *p* < 0.001, ƞ2 = 0.14). Similarly, TRACK-MS showed a larger correlation with disability with a large effect size (*r*_TRACK-MS_ = −0.57, CI: −0.75 to −0.39). The BICAMS demonstrated medium-sized correlations (*r*_BICAMS_ = 0.39, CI: −0.57 to −0.21). Furthermore, TRACK-MS had good test–retest reliability (*r*_TRACK-MS_ = 0.86, CI: 0.79 to 0.91), although the BICAMS had a better reliability (*r*_BICAMS_ = 0.92; CI: 0.87 to 0.95). To assess practice effects, we calculated the change in test performance from pretest to posttest. In the HC group, there was no significant improvement from pre- to posttest in the BICAMS (*d* = 0.07, CI: 0.05 to 0.18) and TRACK-MS (*d* = 0.02, CI: −0.13 to 0.17) with neglectable effect sizes. In the total sample, there was a significant improvement from pre- to posttest for the BICAMS (*d* = 0.14; CI: 0.07 to 0.22) but not for TRACK-MS (*d* = 0.08, CI: −0.01 to 0.18).

Additional analysis regarding the correlation between sequence of test administration and sequence of validity ranking (difference MS vs. healthy controls) revealed no association (rho = 0.21; *p* = 0.62). 

## 4. Discussion

In the current exploratory cohort study, we use a bundle of cognitive tests addressing the cognitive domains typically impaired in MS to identify a very brief test battery—TRACK-MS—with test quality criteria that were at least comparable to the gold standard BICAMS. We hope this very brief test battery will increase feasibility of cognitive tracking in routine clinical practice. Our results provide evidence that TRACK-MS—which takes only about 5 min to administer—is a valid and reliable cognitive tracking tool that is less prone to practice effects than the BICAMS. TRACK-MS also includes the COWAT, which demonstrated the highest single-test validity in our study, and the current gold standard single test, the SDMT [61]. To our knowledge, the COWAT has not been used in any previous study to assess MS-related cognitive impairment. Furthermore, we report for the first time that the combined test battery consisting of the COWAT and SDMT was at least as useful as the BICAMS for detecting cognitive impairment in MS patients. Both the COWAT and SDMT pose high demands on processing speed, which is strongly affected by white matter alterations (e.g., hyperintensities and volume). Hence, the favorable test characteristics of TRACK-MS may result from its demands on processing speed in the visual (SDMT) and in the verbal domain (COWAT) [62]. Our results are in line with studies showing high impairment in processing speed in MS patients [63].

In our study, the SDMT had the second-best validity after the COWAT. Our results for the SDMT are in line with previously reported results [45], which showed that the SDMT and BVMT-R reliably differentiated between MS patients and HCs [64,65]. Similarly, earlier research showed that the SDMT and BVMT-R were the most sensitive tests of the Minimal Assessment of Cognitive Function in Multiple Sclerosis (MACFIMS) battery and that these exhibited the largest differences between MS and HCs [11,66]. Previously, it was also reported that MS patients and HC showed differences mainly in the SDMT and BVMT-R but not in the CVLT-II [67]. Two additional validation studies reported that the SDMT and BVMT-R were able to differentiate between MS patients and HC, whereas the CVLT-II could not [68,69]. The SDMT was repeatedly reported to be better than the PASAT in discriminating between MS patients and HC [70,71]. Additionally, the SDMT has been shown to be the most sensitive test in the Brief Repeatable Neuropsychological Battery [72,73], and to have a higher correlation with the EDSS than the PASAT [74]. A moderate-to-large correlation of the EDSS with SDMT (*r* = 0.55), BVMT-R (*r* = 0.54), and CVLT (*r* = 0.40) has been demonstrated [75,76], corresponding with our results.

## 5. Limitations and Future Perspectives

A limitation of the current study is that the combination of SDMT and COWAT (TRACK-MS) was not defined a priori but was selected based on the data analysis, which demonstrated the highest validity of this test combination. Because of this exploratory approach, the study should be replicated in a validation study. Here, we could only show the lower boundary of practice effects, as the cognitive ability of the MS group probably declined over time. It would be desirable in future studies to assess the real magnitude of the practice effect, thereby allowing for longitudinal correction of scores. Practice effects are difficult to estimate: they likely depend on baseline performance as a result of the regression-to-the-mean effect and the ceiling phenomenon; i.e., individuals with higher baseline performances are likely to profit less from retesting and therefore tent to show smaller practice effects. Because patients with MS tend to have lower baseline scores, this phenomenon likely leads to larger practice effects in patients with MS. This phenomenon was seen in our study, in which larger practice effects were observed in the total sample (BICAMS: *d* = 0.14 vs. TRACK-MS: *d* = 0.08) than in the healthy control sample (BICAMS: *d* = 0.07 vs. TRACK-MS: *d* = 0.02). The development of alternative versions of the COWAT and SDMT could minimize practice effects. It should be pointed out, however, that we provide evidence that the BICAMS has larger practice effects than TRACK-MS.

In terms of the study population, the mean years of education differed in the MS and HC groups at baseline. However, we accounted for this covariate statistically. It should also be noted that the presence of dysarthria may affect the COWAT and SDMT scores [77]. Theoretically, in patients with severe dysarthria, these tests would not measure cognitive flexibility but motoric speech impairment. However, in our study, there were no patients with marked motor disabilities or speech disorders.

Validation was restricted to the difference between patients with MS and HC and the association of the tests with MS-related disability. Links between cognitive scores and brain biomarkers (e.g., inflammatory markers or extent of white matter hyperintensities) are also of interest and could be assessed in future studies. The relatively small sample size resulted in a fairly large confidence interval. Study replication within a larger sample would be expected to increase the precision of the estimates.

## 6. Conclusions and Implications

The data from our study provide evidence that TRACK-MS, a very brief cognitive tracking tool for MS, has at least comparable test quality criteria to the current gold standard, the BICAMS. TRACK-MS can be administered in 5 min and showed better validity and lower practice effects than the BICAMS as well as good test–retest reliability. TRACK-MS is not intended to create a comprehensive profile of cognitive strengths and weaknesses, but to track MS-related cognitive impairment. TRACK-MS poses strong demands on processing speed in the visual and verbal domain. These demands may account for its high validity in assessing MS-related impairment. We report for the first time that the combination of the COWAT and SDMT scores is as useful as the BICAMS in detecting cognitive impairment in MS patients. A larger study to confirm our results would be useful. The TRACK-MS may prove to become a valuable tool in routine clinical practice, allowing time-efficient assessment and longitudinal tracking of cognition in MS patients. Such a reliable assessment tool will allow detection of subtle cognitive impairment, with the hope that early detection may facitilitate the use of therapeutic interventions to positively impact patients’ quality of life. 

## Figures and Tables

**Table 1:** Demographics and clinical characteristics of both groups (MS; healthy controls).

Descriptive Variables	Patients with MS (*n* = 66)	Healthy Controls (*n* = 22)	*p*-Value
Age in years (Mean, SD)	48.3 (10.3)	49.8 (14.8)	0.60 **^a^**
Sex (female/male)	41/25	11/11	0.31 ^b^
Education years (Mean, SD)	10.5 (2.4)	11.8 (2.2)	0.03 **^a^**
EDSS 0–3.0 (*n*) EDSS 3–6.0 (*n*) EDSS > 6.5 (*n*)	22 24 20		
No therapy (*n*)	26		
First line therapy (*n*)	18		
Second line therapy (*n*)	7		
Third line therapy (*n*)	9		
Biotin (*n*)	6		

^a^*t*-test for comparing independent means; ^b^ chi-square test of independence. SD: standard deviation; N: number; EDSS: Expanded Disability Status Scale; MS: multiple sclerosis; First line therapies: interferons, glatiramer acetate, teriflunomide, dimethyl fumarate; Second line therapies: fingolimod, siponimod, cladribine; Third line therapies: natalizumab, ocrelizumab.

**Table 2:** Baseline and one year follow up cognitive tests results of MS patients and healthy controls.

Variables	Patients with MS (*n* = 66)		Healthy Controls (*n* = 22)	
	Baseline	1 Year	Baseline	1 Year
Verbal episodic memory VLMT total correct, mean (SD) VLMT delayed recall, mean (SD)				
	51.3 (10.6)	54.4 (10.4)	59.3 (8.4)	63.1 (7.9)
	10.3 (3.3)	11.0 (3.1)	12.8(1.9)	13.5 (1.8)
Visual episodic memory BVMT-R total correct, mean (SD) BVMT-R delayed recall, mean (SD)				
	21.1 (7.5)	22.0 (5.3)	26.4 (5.7)	25.9 (6.5)
	8.3 (2.6)	8.2 (2.0)	10.5 (1.6)	9.6 (1.7)
Verbal attention-executive functions COWAT, mean (SD) PASAT-3 total correct, mean (SD)				
	28.7 (9.4)	29.7 (9.1)	39.5 (7.3)	40.5 (7.2)
	41.3 (13.8)	43.5 (12.2)	47.9 (6.3)	47.8 (7.4)
Visual attention-executive functions SDMT total correct, mean (SD) BDT, mean (SD)				
	46.0 (13.6)	48.1 (14.0)	56.4 (8.2)	55.5 (9.3)
	43.1 (11.4)	45.9 (11.4)	52.0(10.2)	52.4 (9.4)

SD: Standard Deviation; MS: multiple sclerosis, VLMT: Verbal Learning and Memory Test; BVMT-R: Brief Visual Memory Test-Revised; COWAT: Controlled Oral Word Association Test; SDMT: Symbol Digit Modalities Test; PASAT-3: Paced Auditory Serial Addition Test-3 s Version; BDT: Block Design Test.

## Data Availability

The data that support the findings of this study are available on request from the corresponding author, [DT], upon reasonable request.

## Supplementary Materials

The following supporting information can be downloaded at: https://www.mdpi.com/article/10.3390/biomedicines10112975/s1; Description of the psychometric tests. The cognitive tests are described in Taranu et al, 2021 [60], as well.
