# Supplementary material for: The TRACK-MS Test Battery: A Very Brief Tool to Track Multiple Sclerosis-Related Cognitive Impairment
Source: Biomedicines. 2022 Nov 18;10(11):2975. doi: 10.3390/biomedicines10112975 (PMC9687502; doi:10.3390/biomedicines10112975)
Supplement: Supplementary file 1 [file biomedicines-10-02975-s001.zip › biomedicines-2011772-supplementary.pdf]

biomedicines

Supplement: Test Descriptions

Controlled Oral Word Association Test

The Controlled Oral Word Association Test (COWAT, letters F-A-S) has been used to measure both language and executive function domains.

In this test, the subject should say as many words as possible that begin with the letters F, A, or S (German language). For every letter, the subject has 60 seconds of time. Proper names, numbers or words with different tenses or endings once the root word has been given are excluded from the rating. The letters F, A, and S differ in their frequency in spoken German and thus allow the level of difficulty to be graded. The result is a raw score comprised of the total number of said words.

VLMT- Verbal Learning and Memory Test

The VLMT is used to assess verbal memory encoding, retention, and retrieval.. In the learning phase subjects were read a word list consisting of 15 words five times; after each presentation subjects should recall as many words as possible. After being presented with a distractor list consisting of 15 other words, the initial words have to be recollected freely.

A second part takes place after approximatively 30 minutes. In the meantime, the subject is to take other neuropsychological tests that are non- linguistic (the tests BVMT-R, SDMT, PASAT and BDT were administered at this point). The first list of words is then queried again without being presented again. Finally, a recognizable list consisting of the 30 words from the two lists, as well as 20 additional words is read, and the subject must indicate whether each word came from the initial word list.

The following values are used to assess the verbal memory performance of the test subjects: the reproduced words from the first five rounds are summarized as an overall learning performance value; subjects were able to achieve a maximum score of 75. For the number of correctly reproduced words according to the interference list after a delay, test values

between 0 and 15 are possible: 0 would indicate that no words were correctly recognized and 15 that every word was correctly recognized.

BVMT-R- Brief Visuo-spatial Memory Test-Revised

The BVMT is used to assess visual memory capacity and spatial memory. The test consists of a stimulus sheet on which six independent geometric figures are arranged, and a total of three answer sheets.

The examiner presents the subject the sheet with the 6 figures for ten seconds. Then the sheet is covered. The subject must draw the figures as correctly as possible in the original position. The subject has no maximum time for this task, and decides when the test ends. The answer sheet is collected and the subject receives the second answer sheet in exchange. Another iteration follows in which the stimulus is presented again for 10 seconds and then covered. A total of three iterations are performed. The second part of the test follows after 30 minutes, when the subject has to draw the 6 figures without the stimulus being presented.

Two points are awarded for a figure reproduced in the correct place on the answer sheet and in the correct form. A point is awarded for a correctly reproduced figure in the wrong position. A point is also awarded for a figure with errors that is drawn in the correct position.

SDMT- Symbol Digit Modalities Test

The SDMT records the speed of information processing and appears to be the most sensitive test for this capability. Working memory influences the test result to only small extent and the test-retest learning effect is very low. It is therefore particularly well suited for follow-ups. In this test, a series of geometric figures in a legend is assigned a numerical value from one to nine.

The subject assigns the corresponding numerical values to the figures depicted in the legend and has exactly 90 seconds to complete the task. The final test score corresponds to the number of correctly displayed numerical values. To make sure that the test person has correctly understood the test instructions, a short practice session is carried out before the

actual test in that the first ten symbols on the test sheet are assigned under the supervision of the examiner. If necessary, the examiner should correct the test person at this time. After this short test phase, the actual test begins.

PASAT- Paced Auditory Serial Addition Test

The PASAT is intended to provide a rough assessment of cognitive function. During the test, the patient receives a new number from a tape every 3 seconds. It is the patient's task to add the last two numbers together. To cope with the task, the last named number must be kept in mind during addition in order to prepare for the next addition. Therefore, arithmetic skills and above all working memory and attention (in particular shared attention) are required to perform the test. The patient receives one point for each correct calculation, and a maximum of 60 points can be achieved.

For the VLMT, BVMT-R, SDMT, and PASAT, several versions of the test are provided, which can be used alternately in the course of studies to counteract the effects of test repetition.

Block Design Test

This test is part of several test batteries for intelligence measurement, and assesses spatial perception, visuomotor coordination, and distinction between parts and the whole in abstract visual patterns. The Block Design test includes nine identically shaped red and white patterned cubes and nine different printed sample templates as well as a test protocol for the examiner. Subjects were asked to recreate specified patterns using the cubes. The different patterns consist of two, four, or nine dice, and there are varying time limits allowed for replication. Up to sample number 4, the test subject receives two points on the first correct attempt and one point on the second correct attempt. From sample number 5 to 8, the subject receives 4 points on the first correct attempt. From sample number 9 to 14, the subject receives 4, 5, 6 or 7 points. If the subject solves the puzzle quickly, time bonus points are awarded. Without time bonus points, every correct reproduction from sample 1 to 4 receives 2 points, and from 5 to 14 receives 4 points.

In order to show the test subjects how to proceed, the first sample (sample number 5, the starting pattern) is shown by the examiner. The subject should then reproduce the pattern.

The subject then shown a second pattern (number 6) based on a template. If the subject scored zero or one point in questions 5 and 6, four easier patterns are presented in descending order (patterns 1 through 4) until two consecutive patterns were scored with two points. If this happens, the test continues with pattern 7 and two points are credited for the remaining easier tasks. Subjects who solve patterns 5 and 6 with 2 or more points are not presented the easier patterns 1 to 4 and are simply credited with the highest score for them. The test is terminated when the subject scores zero points on two successive tasks. A maximum of 66 points (with time bonuses) can be achieved.
